# The Gut Microbiome as a Target for the Treatment of Type 2 Diabetes

**DOI:** 10.1007/s11892-018-1020-6

**Published:** 2018-06-21

**Authors:** Ömrüm Aydin, Max Nieuwdorp, Victor Gerdes

**Affiliations:** 1 Department of Internal Medicine, MC Slotervaart, Amsterdam, The Netherlands; 2 Department of Internal Medicine, AMC-UVA, Amsterdam, The Netherlands; 3 Diabetes Center, Department of Internal Medicine, VU University Medical Center, Amsterdam, The Netherlands; 4 Wallenberg Laboratory, University of Gothenberg, Gothenberg, Sweden

**Keywords:** Diabetes, Gut microbiota, Metabolism, Obesity, Personalized medicine

## Abstract

**Purpose of Review:**

The objective of this review is to critically assess the contributing role of the gut microbiota in human obesity and type 2 diabetes (T2D).

**Recent Findings:**

Experiments in animal and human studies have produced growing evidence for the causality of the gut microbiome in developing obesity and T2D. The introduction of high-throughput sequencing technologies has provided novel insight into the interpersonal differences in microbiome composition and function.

**Summary:**

The intestinal microbiota is known to be associated with metabolic syndrome and related comorbidities. Associated diseases including obesity, T2D, and fatty liver disease (NAFLD/NASH) all seem to be linked to altered microbial composition; however, causality has not been proven yet. Elucidating the potential causal and personalized role of the human gut microbiota in obesity and T2D is highly prioritized.

## Introduction

The current obesity pandemic is increasing worldwide, thus contributing to the rising incidence and prevalence of type 2 diabetes (T2D) mellitus. Particularly alarming is the equal trend of increase in obesity in children [1, 2]. T2D, notorious for its macrovascular and microvascular complications, presents a heavy challenge for health care systems [3]. Therefore, researchers globally are attempting to identify new preventive and therapeutic options. Obesity puts people at risk for developing metabolic syndrome, defined by increased waist circumference, hypertension, dyslipidemia, and insulin resistance [3, 4]. It has been clarified that obesity contributes to T2D by decreasing insulin sensitivity in adipose tissue, liver, and skeletal muscle, and subsequently impaired beta-cell function. So, the question arises: if insulin resistance is key in developing T2D, what are the contributing factors in its development? For example, DeFronzo describes a total of eight possible pathophysiological derangements contributing to the development of T2D [5], highlighting the multifactorial aspect of T2D and also suggesting the need of a more personalized approach in recognizing these derangements.

Increasing evidence indicates a fundamental role of excess central adiposity in developing the metabolic syndrome [6]. However, the underlying mechanism is not well understood [7]. For example, it is known that surgical removal of subcutaneous [8] or intra-abdominal [9] adipose tissue does not improve insulin sensitivity in obese human subjects despite weight loss induction. Underscoring the role of systemic factors maintaining insulin resistance, the search continues and the focus has shifted to the gut microbiota and its systemic properties for the past few years. The gut microbiota has attracted more and more attention as an underlying mechanistic driver in obesity and insulin resistance. Based on recent studies, we see an altered gut microbiota in obesity [10] and view the gut microbiota as an environmental factor contributing to adiposity and insulin resistance [11, 12].

The gut microbiota is a collective term for the microbial community in the gut [13], whereas the gut microbiome is defined as the full collection of genes in the gut microbiota [14]. This interest in gut microbiota is not illogical. Even in 460–377 B.C., the father of modern medicine Hippocrates stated his famous quote, “All disease begins in the gut”. The gut microbiome contains an immense diversity of microorganisms, varying from bacteria as well as viruses, fungi, phages, protozoa, and archaea [15–17] all colonizing our adult bodies. Archaea, bacteria and Eukarya encompass the three-domain taxonomy of life [13]. The introduction of molecular techniques using 16S rRNA gene sequencing created phylogenetic information on microbial taxa. In other words, it can be used to distinguish microbial groups in phylotypes yet lacks specificity to describe bacterial species for which more advanced techniques like shotgun metagenomic sequencing are needed [13]. Recent estimates show that our microbiota equal our total number of our somatic and germ cells [18]. The proposed view of our microbiota as a microbial (endocrine) organ living symbiotically inside our gut has led to a new perspective postulating multiple lineages capable of communicating with each other and shaping host immunometabolism in several ways [12]. This intimate co-evolution has led to an interlocked symbiotic relationship, with diverse capacities including the degradation of otherwise indigestible components of our diet, harvesting of energy and nutrients, shaping of the host immune system, maintaining the integrity of the gut mucosal barrier, and xenobiotic metabolism [12, 19–23]. Thus, gut microbiota complement our biology in ways that are mutually beneficial.

Diversity of the gut microbiota is the result of strong host selection and co-evolution, influenced by many factors. It includes thousands of species with a collective genome, the metagenome, which is close to 5 million genes. Our insight into the composition of the gut microbiota is based on culture-based studies and became more precisely characterized with high-throughput sequencing technology [12]. Several studies have shown the presence of microRNA (small non-coding RNA) extracellularly, which could be indicative for diseases such as intestinal malignancies [24, 25]. MicroRNA is a normal compound in feces in mice and humans, with intestinal epithelial cells and Hopx-expressing cells (intestinal stem cells, later derived in Goblet and Paneth cells) as the major source [26]. The concentration of microRNA is inversely correlated with microbial density, suggesting that it is absorbed by microbes and in this way is influencing the bacterial genes [27]. Sequencing technology makes it possible to analyze the intestinal metagenome based on genetic material from stool samples [28]. The development of these culture-independent genomics methods revived interest of microbiota in the etiology of diseases, allowing better understanding of the complexity of the gut microbiota [22, 29]. Much attention has been paid to identify the diverse composition of microbes in the healthy human population, creating the concept for a “personal microbiota” [30]. Despite the interpersonal variation, the gut microbiota maintain a stable relative abundance at operational taxonomic unit levels [31]. Five phyla dominate the microbial community: Actinobacteria, Bacteroidetes, Firmicutes, Proteobacteria, and Verrucomicrobia [16]. From animal studies, we know that rapid luminal flow, high acidity, and secretion of bile acids and other compounds cause a changing microbial density along the gastrointestinal (GI) tract. The gradient starts with low density in the upper GI tract and it expands towards the rectum [32]. The proximal part of the GI tract is enriched with Lactobacillaceae (belonging to *Firmicutes*) and proteobacteria (*Enterobacteriaceae*), whereas the more distal large intestine shows higher concentrations of Firmicutes (*Lachnospiraceae*, *Ruminococcaceae*) and anaerobes such as Bacteroidetes (*Bacteroidaceae*, *Prevotellaceae*, and *Rikenellaceae*). The representation of Verrucomicrobia, *Akkermansia muciniphila*, is also mainly located distally [33–36].

Metagenomic-wide association studies combine metagenomic data with clinical features. Such studies presented significant differences on a metagenomic level between metabolically healthy versus metabolically unhealthy subjects [37, 38]. The Human Microbiome Project (HMP) [30] from the USA as well as LIFELINES [39••], and the Flemish Gut flora cohorts in Europe [40] are the largest cohorts to date (> 1000 subjects), containing high-throughput metagenomic data serving as a catalogue of the human microbiota. Although small, there seems to be a relationship between human metabolism and gut microbiota composition in otherwise healthy subjects.

Symbionts and commensals are not the only microorganisms the host encounters. The host immune system is continuously challenged by distinguishing beneficial microbes from pathogens. The interactive role of phages [41] and fungi [42] in gut microbiota composition is also gaining more attention. Although still poorly understood, the gut microbiota and the host immune system have co-evolved so profoundly that they can influence our immunological well-being [17]. This is seen in germ-free mice, where the absence of a microbiota leads to defects in the development and function of the immune system [43]. This described dynamic maturation of the gut microbiome and host immune system is determining host-microbe interactions and influencing the susceptibility to infection, inflammatory diseases, and autoimmunity [21].

**Fig. 1:**
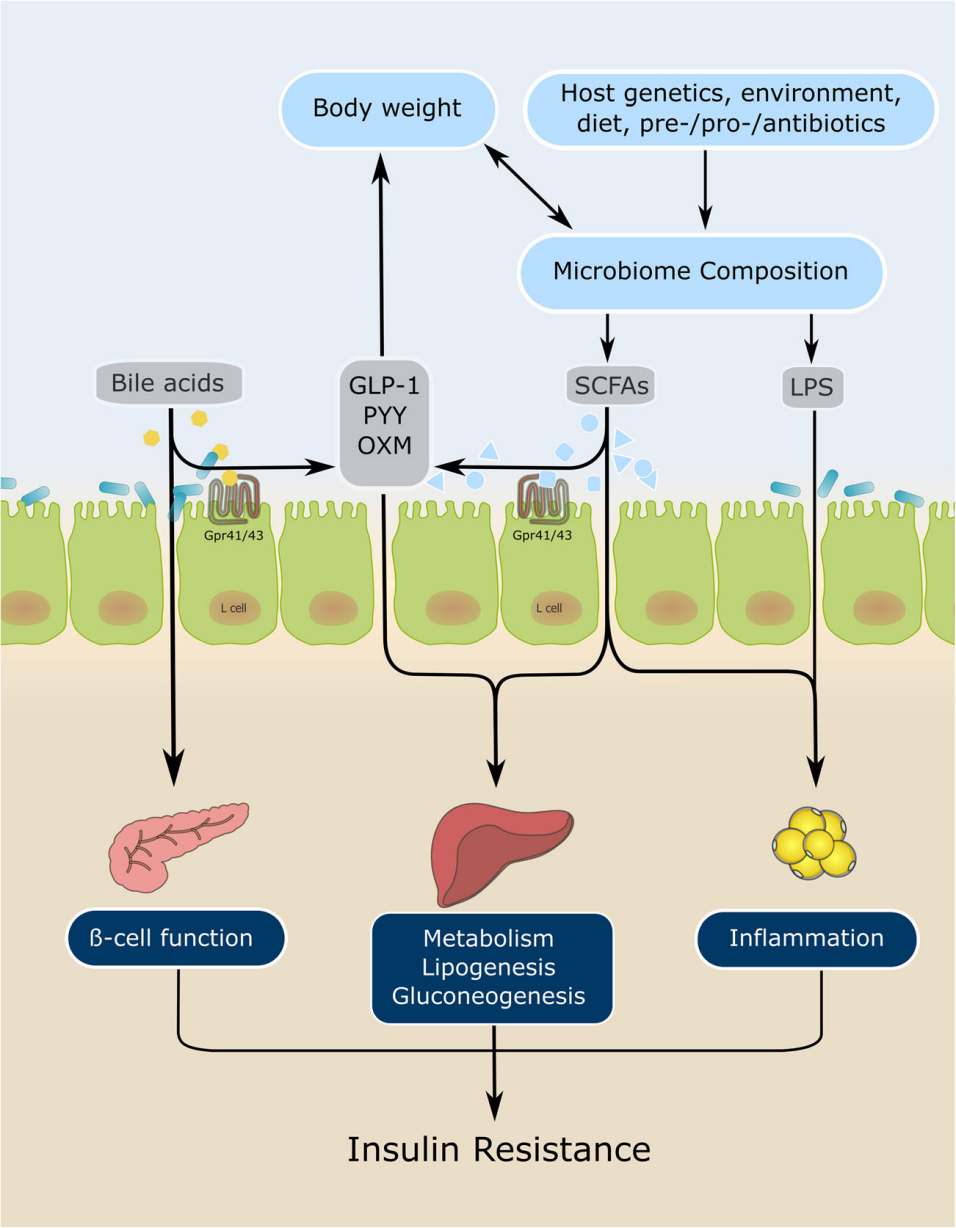
Effect of gut microbiota in liver disease, insulin resistance, and type 2 diabetes. GLP-1, glucagon like peptide 1; Gpr41, G-coupled receptor 41; Gpr43, G-coupled receptor 43; LPS, lipopolysaccharides; OXM, oxyntomodulin; PYY, protein YY; SCFA, short-chain fatty acids

There are different factors influencing the development of the microbiome in the early years of life, starting with the mode of birth [44, 45], breastfeeding or formula-feeding infants, and possibly the introduction of solid food [46]. The intestinal microbiome stabilizes about 3 years after birth, when it resembles the adult microbiome and stays relatively stable over time [47, 48]. In adulthood, the microbiome can be altered by changes in diet [49] as well as by the use of several types of medication such as antibiotics [50], metformin [51, 52], and even proton pump inhibitors [53]; this is also illustrated in Fig. 1. Nonetheless, by deep sequencing the gut microbiomes in a large Caucasian cohort, only 18.7% of the interpersonal variation in microbial composition could be explained by host characteristics (physiologic and biomedical measures), previous diseases, medication use, smoking, and dietary factors [54].

## The Gut Microbiota in Cardiometabolism

Diet is a modulator of the composition and the function of the gut microbiota [20]. Human intervention studies have revealed some aspects in which diet can alter the gut microbiota. First, the microbiota alters rapidly when exposed to great and fast changes in diet. Short-term dietary changes such as switching between plant- and meat-based diets, or adding more than 30 grams of fiber per day to the diet, or following a diet with a different fat/fiber content can change the human gut microbiota in function and composition significantly in 48 hours [49, 55, 56]. Fiber-enriched diets have been shown to improve insulin resistance in lean and in obese subjects with diabetes [57, 58]. However, only long-term dietary habits are most important in actually shaping the composition of the gut microbiota. Short-term dietary interventions failed to change the major features and classification of the microbiota [55]. Another aspect to consider is the high interpersonal variance in the effect of changed diets on the gut flora, thus mirroring the individualized nature of our gut microbiota [20]. Moreover, the landmark paper of Zeevi et al. elucidated the possibility of predicting individual glycemic responses to diet based on the composition and function of the individual microbiota. They measured continuous (postprandial) glycemic responses to meals during 1 week in a human cohort of 900 subjects and found high interpersonal variability to identical meals. Using machine learning algorithms, the authors were able to predict post-prandial responses to meals. Also, they effectively used and validated these algorithms to personalize dietary recommendations [59•].

In line with their finding that diet is an important driver of gut microbiota composition, Yatsunenko et al. found that gut microbiomes differ over human populations and that Malawian and Amerindian infants have more shared features than when compared to those of American infants [60].

It is thought that (aromatic) metabolites derived from gut microbiota processing of dietary compounds are important factors influencing host physiology; conversely, the responses to nutrients are influenced by the gut microbiota. Choline and trimethylamine-N-oxide (TMAO) are two of those impacting micronutrients. For example, Spencer et al. showed that a choline-deficient diet in humans modulates the gut bacteria with altered levels of *Gammaproteobacteria* and *Erysipelotrichi* and this is directly associated with fatty liver development [61]. Wang et al*.* experimented in atherosclerosis-prone mice, supplementing a diet with either choline or TMAO, which resulted in increased levels of TMAO in plasma; the latter enhanced atherosclerotic plaques. The previous link was negated in atherosclerosis-prone mice with antibiotic-suppressed microbiota [62], underscoring the relationship between the gut microbiota, the consumed diet, and host metabolism.

Several studies, mostly animal models, demonstrated the microbiota as a major factor in the development of obesity [10, 11, 63, 64]. This is illustrated in a study on genetically obese ob/ob mice, which had a 50% reduction of *Bacteroidetes* and an increase of *Firmicutes* in comparison to their lean siblings [10]. These differences affect the metabolic potential of the gut microbiota, and these ob/ob mice are known to have an increased capacity in harvesting energy from ingested food. Furthermore, this highly sufficient harvesting of energy is a trait which appeared to be transmissible to germ-free mice through microbiota transplantation resulting in increase of obesity [63].

A similar composition of the microbiota is seen in obese human subjects, and the microbial ecology in humans changes with weight loss [65]. The transmissible trait of the gut microbiota has also been reported in humans. A fascinating study by Ridaura et al. showed that transferred human microbiota from discordant obese twins into mice changed their phenotype, underscoring potential causality. Transplanting the gut microbiota of the human obese twin significantly increased adipose mass in the recipient mice, and this effect was reproducible. They measured increased butyrate and propionate levels in mice colonized with lean human gut microbiota, compared to obese human gut microbiota. [66]. This demonstrates that not only obesity in mice but also obesity in humans could be transmitted through fecal transplantation. We have also demonstrated that performing a fecal microbiota transplantation (FMT) from lean human donors to obese human recipients with metabolic syndrome improved insulin sensitivity [67]. Influencing the gut microbiota by treatment with probiotics containing *Lactobacillus gasseri* demonstrated positive effects on body weight in overweight and obese subjects treated in a double-blind randomized controlled intervention trial [68]. This also suggests that interventions in microbial composition may have impact on body weight.

Currently, the most effective treatment for obesity in the long term is bariatric surgery [69]. Intriguingly, when mainly focused on the Roux-en-Y gastric bypass (RYGB), the effects of bariatric surgery exceed weight loss alone, with favorable changes in beta cell function in patients with T2D [70]. The metabolic benefits are reported in a few days post-bariatric surgery, which suggest a weight-independent effect [70]. This effect is possibly explained by stimulating the entero-insular axis, with altered levels in GLP-1, PYY, OXM, and ghrelin. GLP-1, PYY, and OXM are all produced by enteroendocrine L cells in the distal small intestine [70]. These intestine-derived hormones have anorexic properties such as decreased hunger, decreased food intake, and delayed gastric emptying. Levels of ghrelin, a gastric hormone known for its orexigenic properties, are reported to be suppressed after bariatric surgery [71]. Moreover, these endogenous peptides are involved in the regulation of insulin sensitivity and insulin secretion.

## Metabolic Action of Gut Microbiota-Derived Short-Chain Fatty Acids

Diet is a major source of substrates for the production of small molecules by the gut microbiota. After absorption, these molecules may have a direct effect on hepatocytes via uptake and transportation in the portal vein. After escaping first-pass metabolism in the liver, these small molecules circulate systemically, and in this way, may contribute to diverse effects on host physiology [22]. Dependent on the substrate (amino acids, lipids, carbohydrates, iron) presented to the intestinal lumen, gut microbes can generate specific (aromatic) metabolites. Most of the otherwise not digestible carbohydrates are fermented by the microbial community to produce short-chain fatty acids (SCFA), of which acetate, propionate, lactate, and butyrate are the most important [72]. The subsequent host response is dependent on the relative proportion of SCFA and subsequent liver clearance, where they induce de novo hepatic lipogenesis and other processes, while only a small subset enters the systemic circulation [11]. It should be highlighted that not all SCFA have similar metabolic effects [73]. The causal role of intestinal bacterial strains in the production of SCFA has been demonstrated clearly using oral antibiotic treatment that induced profound effects on the production of most of these metabolites by the gut microbiota [74]. The importance of iron as a substrate was demonstrated in rats: iron deficiency was correlated with lower levels of cecal SCFA, such as propionate and butyrate, when compared to iron sufficient rats. An increased abundance of *Lactobacilli* and *Enterobacteriaceae* was observed in addition to a significantly decreased abundance of the butyrate-producing *Roseburia* spp./*E*. *rectale* group in iron-deficient rats. Repletion of iron significantly increased cecal butyrate concentrations [75]. SCFA account for approximately 5–10% of the energy source of the body [76]. In this regard, butyrate is known as a key energy substrate for both colonocytes and enterocytes and has been described to play a role in energy expenditure by stimulating mitochondrial respiration and also increasing fatty acid oxidation [77, 78]. On the other hand, acetate functions as a substrate for cholesterol synthesis [79]. Indeed, composition of the gut microbiota and proportions of SCFA are correlated with obesity: obese mouse models show increased propionate levels compared to lean mice with an altered phyla ratio of *Bacteroidetes*-to-*Firmicutes* as well as an increased proportion of *Bacteroidetes* [79]. Conversely, oral administration of butyrate has also been reported to improve insulin sensitivity [78]. It is thought that SCFA are capable of functioning as signaling molecules by binding to G-protein-coupled receptors Gpr41 (FFAR3) and Gpr43 (FFAR2), and thus fine-tune metabolism. [80, 81] Moreover, Gpr43-deficient mice become obese when fed a normal diet, whereas mice with overexpression of Gpr43 remain lean even when consuming a high-fat diet. It was demonstrated that SCFA-mediated activation of Gpr43 led to suppression of insulin signaling in adipose tissue of these mice [82].

Finally, Gpr43 activation by SCFA in (small) intestinal enteroendocrine L cells induces GLP-1 release that can also affect insulin sensitivity via incretin response [83]. In conclusion, intestinally derived SCFA are an important source for energy, but are also capable of acting as signaling molecules in adipose tissue, thereby exerting an influence on energy homeostasis.

## Gut Microbiome in Insulin Resistance and Type 2 Diabetes

As described above, the intestinal microbiota should not be viewed as an isolated community. Instead, it functions as a large metacommunity, in which microorganisms reciprocally affect each other. In this way, they can disperse and colonize new gut habitats [84]. The importance of diversity in metabolic control was shown in landmark papers by Le Chatelier et al. and Cotillard et al. who showed that low diversity in the gut microbiome associates with obesity and a higher prevalence of insulin resistance, non-alcoholic fatty liver disease (NAFLD), and low-grade inflammation [85, 86]. Furthermore, low bacterial diversity was characterized by pro-inflammatory properties, suggested by the reduction in butyrate-producing bacteria and the increase in mucin-degrading bacteria. These characteristics potentially impair the gut integrity causing low-grade inflammation through endotoxemia [76, 85, 86].

As described above, low-grade inflammation of visceral adipose tissue may provide a link between obesity and insulin resistance [87]. Adipose macrophage accumulation in crown-like structures correlates with altered gene expression involved in inflammatory control [88]. These findings indicate a profound role of the innate immune system in insulin resistance. Low-grade inflammation related to increased (translation of) Gram-negative bacterial strains has also been observed in patients with T2D. Mice and humans with diabetes have elevated plasma levels of lipopolysaccharides (LPS), a bacterial endotoxin derived from the membrane of Gram-negative bacteria. This phenomenon, also known as metabolic endotoxemia, is induced via either bacterial translocation over the intestinal wall or partly via bacterial capsule fragments that enter the bloodstream. Indeed, increased levels of circulating LPS have been shown to impair glucose metabolism in mice [89, 90]. To underscore the relevance to humans, Qin et al. used the concept of metagenomic linkage groups (MLG) and observed an abundance of *Escherichia coli* in the T2D MLG, a bacterial strain that is thought to be one of the major sources of LPS in humans [38]. LPS and other gut-derived molecules, such as peptidoglycans [91] and flagellins [92], activate pattern recognition receptors at the surface of immune cells, such as Toll-like receptors, triggering the production of pro-inflammatory cytokines [93].

Microbiome sequencing also revealed that butyrate-producing Clostridialis (*Roseburia* and *Faecalibacterium prausnitzii*), known for their anti-inflammatory properties [67], are less abundant in the feces of subjects with T2D compared to controls [37, 38]. In contrast, animal data that showed that *Akkermansia muciniphila* was associated with improved metabolic control and less obesity in mouse models of T2D [94]. Qin et al. reported an abundance of *Akkermansia muciniphila* in the T2D MLG in humans [38]. These findings potentially underscore the differences between human and mouse models of metabolism in relation to microbiota composition, but may also be explained by sampling errors because stools that have been exposed to the epithelial mucus layer are more likely to comprise *Akkermansia muciniphila.* Moreover, ethnic differences between human populations may also affect microbiota composition. Karlsson et al*.* compared data of T2D-associated metagenomes between Chinese and Swedish subjects with T2D, which indicated that different intestinal bacterial species were involved in similar metabolic functions [37]. The authors were also able to distinguish subjects with T2D from healthy subjects, with a predictive power exceeding that of body mass index (BMI) [37]. Nevertheless, these data should be interpreted with caution. Finucane et al*.* performed a meta-analysis, pooling results from four studies, including publicly available data from HMP and MetaHIT. This meta-analysis confirmed the differences in phylum-level taxonomic composition between lean and obese subjects. However, it could not reproduce the association between BMI and taxonomic composition [95]. Composition of the gut microbiota does not always translate into function. Identifying the microbiota-derived metabolites through metabolomics gives new insight into the functional capacity of the gut microbiota. Pedersen et al*.* performed metabolomics and microbiome profiling in subjects with T2D and without diabetes. They reported increased plasma levels of branched-chain amino acids (BCAA) as characteristic of individuals with insulin resistance, which in turn correlates with specific bacterial species (*P. copri* and *B. vulgatus*) in their gut microbiota [96]. Systems biology approaches can be used to connect microbial composition to functional capacity. The latter has been extensively reviewed by Meijnikman et al*.* [97].

In conclusion, in the past decade, animal and human studies have identified relevant differences in intestinal microbiota composition in subjects with obesity and T2D [10, 89, 98]. However, the causality and magnitude of effect on metabolic function remains to be proven [99].

## Gut Microbiota in Lipid Metabolism

In recent decades, it has become clear that many metabolic, inflammatory, and innate immune mechanisms are also coordinated by (dietary-derived) lipids [4]. The nutritional importance of dietary lipids is unequivocal, but they also operate as (proinflammatory) ligands binding to nuclear receptors [100]. These include peroxisome-proliferator-activated receptor (PPAR) and liver X receptor (LXR) families, which are pivotal in metabolic and inflammatory pathways. Numerous fatty acids are capable of activating all three members of the PPAR family, thereby improving insulin action and suppressing production of pro-inflammatory cytokines such as TNF-α [101, 102].

G-coupled protein receptors (Gpr) are also activated by both diet-derived metabolites and lipids. For example, the activation of Gpr43 by the dietary-derived metabolite acetate directly reduces lipolysis in adipocytes leading to decreased plasma-free fatty acid levels. This suggests a potential therapeutic role for Gpr43 in the regulation of lipid metabolism [103].

Lipid accumulation in conjunction with low-grade inflammation is a pathophysiological hallmark of atherosclerosis [3]. There is emerging evidence that the pathophysiology of atherosclerosis is related to interpersonal gut microbiome differences [104]. Atherosclerosis seems to be related to TMAO, which is a new marker associated with increased risk of atherosclerosis and coronary artery disease [62]. Human studies using short-term antibiotic intervention have shown that changes in the composition of gut microbiota drives differential production of dietary choline and L-carnitine that can be converted by the gut microbiota to trimethylamine (TMA), which is subsequently oxidized by the hepatic flavin monooxygenases to form TMAO [105]. A major source of dietary choline is food rich in lipid phosphatidylcholine, also known as lecithin, which is present in milk, eggs, liver, red meat, poultry, and fish [62]. However, causality has not yet been established, and our recent fecal transplantation study failed to show altered TMAO production in humans with increased CVD risk that underwent donor FMT [106].

Other key intestinal regulators of lipid and cholesterol metabolism are bile acids, which are involved in facilitating intestinal absorption and transport of diet-derived nutrients, vitamins, and lipids. Whereas bile production takes place in the liver (and is facilitated by products derived from lipid catabolism), 95% of all bile acids will be reabsorbed in the terminal ileum and subsequently re-absorbed by the liver, constituting the so-called enterohepatic circulation. The intestinal microbiota is responsible for converting primary bile salt to secondary bile salts via bile acid de-hydroxylation [107]. Although short courses of oral antibiotics affect intestinal microbiota composition and bile acid metabolism in humans, we found differential effects on glucose metabolism [108, 109]. A more drastic intervention in the intestinal physiology is the RYGB which also has a major influence on enterohepatic bile acid circulation. For example, increased concentrations of primary plasma bile acids have been reported after RYGB, while this effect has not been seen after adjustable gastric banding [110, 111]. In murine models, bile acids promote the release of (enteroendocrine L-cell produced) GLP-1 through the activation of Takeda G-protein coupled receptor-5 (TGR5), thus affecting insulin secretion and whole-body insulin sensitivity [112]. Another important receptor for these bile acids is the farnesoid X receptor (FXR), which is mainly expressed in the liver and intestine, but also in pancreatic beta cells [113]. However, some studies could not find elevated plasma bile acid levels in the first days after bariatric surgery, when most of the metabolic benefits are seen [114, 115]. Thus, bile acids might contribute to the long-term metabolic benefits of bariatric surgery by improving intestinal hormone secretion, but do not explain the observed metabolic effects immediately after surgery.

## Gut Microbiome in Appetite

Obesity is defined as an imbalance between energy intake (usually food intake) and energy expenditure [116]. The brain is a key regulator in detecting alterations in energy balance and induces behavioral and metabolic responses to correct these alterations. The hypothalamus plays an important role in regulation of both food intake as well as energy homeostasis, receiving hormonal and (vagal) neuronal information from the periphery [117, 118]. The gut-brain axis is increasingly highlighted as an important pathway connecting gut microbiota and its metabolites with central regulation of metabolism. For example, in a recent study, mice were exposed to a high-fat diet with SCFA butyrate, either intragastric or administered via intravenous injection. Interestingly, intragastric administration reduced food intake by 21% in 24 hours, while intravenous injection did not lead to any behavioral changes in food intake, suggesting that the effect of butyrate on feeding behavior is indirect through a mechanism involving gut-brain neural circuits [119].

SCFA has also been implicated in intestinal gluconeogenesis (IGN), which is thought to influence food intake and glucose metabolism in mice [120]. Recent animal work showed that SCFA such as butyrate and proprionate might exert part of their beneficial metabolic effects via induction of IGN and IGN genes. De Vadder et al. suggest direct activation of IGN genes in enterocytes by butyrate, while proprionate stimulates Gpr-41 in the periportal afferent neural system and indirectly activates IGN via a gut-brain communication axis [121]. However, this effect has not been reproduced in humans.

As seen in animal models, IGN induces release of glucose into the portal vein where glucose sensors are located and these sensors transmit signals to the brain by the peripheral nervous system, which curbs hunger and improves insulin resistance [122].

Animal studies describe IGN as an important factor in metabolic control both after protein-enriched diet [123–125], and after RYGB surgery [126]. Troy et al. presented a hepatoportal sensor pathway providing feedback to IGN regulation as a driving mechanism for the beneficial insulin-sensitizing effects after gastric bypass in mouse models [126]. On the other hand, a recent study could not detect significant differences in glucose levels between the portal venous blood and central venous blood during RYGB surgery and 6 days after surgery in human subjects with T2D [127].

Changing the gut microbiome composition with prebiotics has also been shown to affect portal vein levels of other hormones including GLP-1, which in turn affected food intake, followed by a decrease in body weight and fat mass [128]. This supports the hypothesis of the communicating property of the gut-brain axis. Experiments with prebiotic-treated mice demonstrated an increase in GLP-1 in portal venous blood, whereas the orexigenic hormone ghrelin was decreased in plasma [128]. Supporting the latter, GLP-1 receptor knock-out mice are completely insensitive to the beneficial effects of prebiotic treatment [129, 130]. This effect has also been demonstrated in humans treated with prebiotics, wherein peripheral plasma changes in GLP-1 and PYY levels were associated with increased satiety and decreased hunger scores [131]. As discussed above, Gpr41 and Gpr43 are also expressed on enteroendocrine L cells, which makes is plausible to consider a possible link between Gpr41 and Gpr43 activation on L cells by SCFA, thereby inducing secretion of GLP-1 and PYY [83, 130, 132]. Taken together, the gut microbiota is perfectly capable of interacting with other organs, such as the brain, potentially functioning as an individual endocrine organ and using metabolites and hormones as key messengers.

## The Gut Microbiome: Conclusions and Future Perspective

The studies discussed in this review suggest that individuals who are obese are likely to have an imbalance in gut microbiota composition and a ‘gut signature’ varying with metabolic control [76]. This possibility is an exciting avenue for further research and possible novel treatment targets. However, because most studies have been undertaken in animals, direct translation of the findings to human is limited. Many studies have been carried out in animal models, which differ significantly in metabolism, gut microbiota composition, and immune system [133].

Moreover, conclusions on the magnitude and causal role of the intestinal microbiota in human metabolism are blurred by other confounding factors, such as (ethnic) differences in study populations, differences in sequencing and analytical techniques, and variability of diets used for the studies. Therefore, guidelines for standardized techniques are essential.

Shifting towards large prospective cohort studies will provide us with more evidence and information on clinical relevance. Similarly, mapping patients with pre-diabetes and diabetes, and observing their diet, type of obesity, metabolic parameters, medication, and inflammatory state is essential for clarifying whether microbiome composition and the presence of metabolites is a result of disease phenotype or if it is causally interlocked with the underlying pathophysiology. Identifying the most important microbiota-related metabolic pathways will enable us to develop new therapeutic agents by influencing these microbiota-related pathways.

Treatment with prebiotics or with newly identified beneficial bacterial strains are potential interventions that will be used in the near future, and it will be important to evaluate their efficacy. Likewise, interventional studies with metabolites of microbiota will be performed (including SCFA butyrate supplementation) to evaluate if this compound has similar effects on food intake, energy expenditure, and improved metabolic features in humans [119].

Newly identified bacterial strains with probiotic potential will probably be found using FMT. For example, our recent study illustrated how donor FMT can (transiently) improve insulin sensitivity in male recipients with metabolic syndrome, bringing new insight in pathophysiology [134••]. Nevertheless, FMT procedures need standardization to provide safety and similar quality between centers. It should also be considered that the therapeutic effect from FMT is not only from bacteria; other microorganisms or other features from the donor could enhance the observed effect during FMT [135].

In conclusion, the modifiable effects of the human gut microbiota on the development of metabolic syndrome make its manipulation a promising therapeutic approach. Analyzing and mapping individual microbial composition on a metagenomic level provides insight into specific targets for treatment and contributes to personalized therapeutic interventions.
